# Why health systems cannot fix problems caused by food systems: a call to integrate accountability for obesity into food systems policy

**DOI:** 10.1017/S1368980024001848

**Published:** 2024-11-07

**Authors:** Erica Reeve, Penny Farrell, Anne Marie Thow, Senoveva Mauli, Dori Patay

**Affiliations:** 1 Global Centre for Preventive Health and Nutrition, Institute for Health Transformation, School of Health and Social Development, Deakin University, 1 Gheringhap Street, Geelong, VIC 3220, Australia; 2 Menzies Centre for Health Policy and Economics, Charles Perkins Centre (D17), Sydney School of Public Health, The University of Sydney, Sydney, NSW 2006, Australia; 3 Australian National Centre for Ocean Resources and Security, University of Wollongong, Wollongong, NSW 2522, Australia

**Keywords:** Food systems, Obesity prevention, Accountability, Public policy, Prevention

## Abstract

Overweight and obesity now impact one-third of the entire adult population globally, and play a role in the development of 3 of the 4 more common causes of death. Accountability systems for obesity prevention centring on food environment policies and health system strengthening have been vital for raising awareness to the lack of progress in prevention. However, health systems have struggled to prevent and treat obesity – in part because critical food systems reforms largely lay outside the mandate of health sectors and with government agencies for agriculture, industry, infrastructure, trade and investment, and finance. In this commentary we highlight aspects of food systems that are driving poor diets and obesity, and demonstrate a powerful but largely overlooked opportunity for accountability mechanisms for obesity that better address food systems as a main driver. We draw on lessons generated in the Pacific Islands Region where they have demonstrated remarkable commitment to obesity prevention through food system reforms, and the adoption of accountability systems that bring leaders to account on these. We make recommendations for accountability mechanisms that facilitate greater cooperation of food systems sectors on obesity and NCD prevention.

Unhealthy diets are one of the leading causes of mortality worldwide, contributing to 1 in 5 deaths every year (2017)^(1)^. Unhealthy diets have been the main driver of increasing rates of overweight and obesity^(2)^, in more than 70 countries of the world prevalence of overweight and obesity has more than doubled since 1980, and it has risen more than tenfold in children and adolescents^(3,4)^. Overweight and obesity now impact one-third of the entire adult population globally^(3)^, playing a role in the development of 3 of the 4 more common causes of death (CVD, cancer and diabetes), with impacts on health and productivity equalling over US$2 trillion per year (€1·86 trillion) (2014)^(2,5)^. This impact is greater in low- and middle-income countries where unhealthy diets have transitioned more quickly^(6)^, and health system resources to manage the health impacts of obesity and noncommunicable diseases (NCDs) are more scarce^(7)^.

Despite good evidence on the actions that should be taken to address overweight and obesity, political support and resourcing to take such actions have not equalled the size of the challenge^(8,9)^. This has led to the introduction of accountability mechanisms that call governments to account for progress against overweight and obesity^(9,10)^. The INFORMAS accountability framework provides a four-step approach for reorienting food environments such that they promote healthier consumption^(9)^. The World Obesity Federation’s Obesity-NCD Preparedness Index scores countries on their ‘readiness’ to address obesity, against indices for health system preparedness, surveillance and reporting, and obesity prevention policies^(11)^. Such accountability systems are vital for tracing progress by governments to stem rising rates of obesity and childhood obesity^(9)^.

Investments in health systems strengthening and universal health coverage to *treat* NCDs are key for identifying health inequities and managing poor health, but there is an opportunity for systems-based *prevention* that is missing. As well as *health system* preparedness, and the *promotion* of healthy food environments, for which we see important momentum with respects to accountability, there is a powerful but largely overlooked opportunity for accountability mechanisms that better address *food systems* as a driver of unhealthy diets, and consequently obesity and NCDs.

## Food systems as a main driver for obesity

Food systems encompass all activities involving the production, harvest, processing, transport, trade and consumption of food via the food environment^(2)^, and as such are a major driver of unhealthy diets^(2,6)^. Dramatic shifts in the availability, convenience and affordability of unhealthy, processed, often packaged food, along with increasing costs and decreased accessibility and availability of fresh, nutrient-dense food have been the main drivers of obesity and diet-related disease globally^(12,13)^.

We highlight three interlinked aspects of food systems that are driving poor diets and obesity, noting these sit well outside of the purview of the health sector. First, agricultural subsidies globally have tended to favour a small number of energy-dense commodities, including refined wheat, rice, sugar, vegetable oils, red meat and poultry^(14)^, with limited capital investment in aspects of food systems critical to the production and supply of nutrient-dense foods such as fruit and vegetables, fish and other aquatic foods^(13,14)^. Second, modernising food systems have aided the large-scale production, supply and retail of ultra-processed and long-life foods, which have had their flavour and stability enhanced by the addition of salt, hydrogenated vegetable fats and sugar and now make a substantial contribution to diets worldwide^(12,15)^. Third, in pursuit of growth and expansion, food companies have rapidly expanded into emerging markets, aided by aggressive marketing campaigns and domestic food takeovers, and increased trade liberalization ^(12)^.

## Effective accountability for obesity must hold to account food systems

Health systems have struggled to prevent and treat obesity – in part because critical food systems reforms largely lay outside the mandate of health sectors and with government agencies responsible for agriculture, industry, infrastructure, trade and investment, and finance. These agencies are likely to hold greater priority for productivity, value addition, export and food sufficiency, goals that can at times be in conflict with health sector aims for improving population nutrition and NCD prevention^(16)^.

Accountability in food systems sectors most often remains oriented towards the production of food for economic development and trade, and not toward assuring an affordable, desirable, accessible healthy food supply^(16)^. At the same time, accountability systems for obesity centre largely on health sector-led initiatives, perpetuating the notion that obesity is ‘health’s responsibility’, and that it can be addressed through health systems actions.

Such notions are being reinforced in the development agenda. For example, the accountability framework for Australia’s Department of Foreign Affairs and Trade (DFAT) new International Development Policy will report impacts in health against ‘coverage of essential services and Universal Health Care (UHC) Ratings^(17)^, in lieu of any measures for diet-related disease prevention, despite this being the main cause of ill health in the Indo-Pacific Region, and a major development challenge moving forward^(18)^.

There is thus a need for accountability mechanisms related to action on obesity and NCD prevention that facilitate greater cooperation between the health and food systems sectors. One way to facilitate this would be to adopt integrated accountability systems that reflect and reward the efforts across *both* food environments *and* food systems. Specifically, there is a missed opportunity to recognise effective policy across all the components and activities that make up the food system (food production, processing, manufacturing, distribution, trade and acquisition) – and point out the longstanding gaps. The Global Food Systems Dashboard enables cross-country comparison of food systems drivers and outcomes at a very high level (e.g. obesity rates, affordability of healthy diets, food losses), but these must be complemented with a more operational set of indices on initiatives to achieve those high-level changes at the country level^(19)^.

In line with global goals to transform food systems, accountability benchmarks for obesity prevention that relate to the food system should reflect efforts by government to first, incentivise the sustainable production of nutrient-rich foods (especially fruit and vegetables) and second, ensure these foods move efficiently across value chains and are widely available and affordable^(20)^. Third, they should reflect concrete measures that disincentivise the supply, marketing and retail of processed foods high in sugar, salt and saturated fats^(20)^, for instance through food taxes and subsidies, regulation of unhealthy food marketing and public food procurement in which healthy choices are the only choice^(9)^. Fourth, accountability to obesity prevention at the national level should measure the impact of these wholesale changes in countries, including food pricing and distribution and dietary quality (e.g. fruit and vegetable consumption, sugar or sodium intake)^(21)^. Operationalising this vision for accountability will require thinking differently about key indicators of success, with oversight mechanisms that institutionalise action on obesity and NCD prevention across the food system.

## Case study: The Pacific experience

The Pacific experience provides an excellent illustration of the opportunity that exists to strengthen food systems’ accountability for obesity prevention. Pacific Island Countries and Territories experience the highest rates of obesity in the world^(11)^, and the well-documented dietary transition towards diets high in refined grains, animal fats, edible oils, sugar-sweetened beverages and processed foods has been a clear driver of obesity and diet-related NCDs^(22)^.

The ranking of many Pacific Island Countries and Territories in the Obesity-NCD Preparedness Ranking indicates that many are falling short in ‘preparedness’^(11)^. This finding is made largely on the basis that many have not yet achieved good health coverage or strong surveillance systems across all levels of governance, and many have not implemented obesity strategies^(11)^. But compared with other settings, Pacific Island Countries and Territories have demonstrated remarkable commitment to obesity prevention and to food system reforms^(18)^. They have been early adopters of taxes on unhealthy foods and beverages, and 75% of Pacific Island Countries and Territories have adopted salt reduction initiatives^(18)^.

The Pacific Islands Region is one of the only regions with annual, region-wide monitoring and accountability against the adoption of a selection of obesity and NCD prevention policies^(18)^. Recognising the role of food systems in the development of obesity and diet-related NCDs, Pacific Island Countries and Territories have begun making concrete steps towards reorienting food systems such that they better promote healthy diets in addition to other environmental and social outcomes via new National Food System Transformation Pathways^(23)^. A regional Pathways document reports progress across a range of food systems concerns (e.g. the triple burden of malnutrition, food governance, food trade and economics, fisheries and agriculture, inclusion and diversity, traditional knowledge, and climate change)^(23)^. Pacific Island Countries and Territories have also begun institutionalising new cross-sectoral governance mechanisms that oversee the implementation and accountability of food systems policies to promote the availability and affordability of a diverse healthy food supply. For example, Vanuatu is in the process of establishing a Food Council positioned under the Prime Minister’s Office that integrates NCD prevention into the new national trade framework. PNG is institutionalising a new National Food Coalition, and regional support agency the Pacific Community (SPC) has recently set up a Food Systems Flagship Programme to promote inter-departmental collaboration within the organisation^(24)^. Though there is still much to do, the commitments of countries in the Pacific Islands Region towards designing and adopting food policies thus represents a significant contribution to obesity preparedness for prevention rather than treatment.

## The challenge ahead

A key challenge for obesity prevention has been that food systems sectors often have very limited ownership of health and nutrition challenges^(25)^ or are held accountable to meeting benchmarks that may conflict with efforts towards obesity prevention; for instance, expanding industry development and trade^(26)^. Engaging food system sectors in obesity prevention will require a major paradigm shift such that they are encouraged by leaders to carry an aligned sense of purpose and responsibility for their role in providing an affordable, desirable, accessible healthy food supply, and then supported towards their commitments. Such shifts will require strong and decisive values-based leadership, platforms for multisectoral partnerships, and supra-sectoral oversight mechanisms that institutionalise action on obesity and NCD prevention into policies across the food system^(16,27)^.

To support this shift, the health policy community can work to draw attention to the relationship between food systems and obesity, and demonstrate where policy actions for obesity prevention have the potential to contribute to other development priorities against which food systems sectors are responsible. For example, taxes on unhealthy packaged foods can reduce consumption of fat, sugar and sodium, and improve the relative affordability of local produce, all while generating revenue that can be invested into the food system to address supply side constraints for local producers^(2,14)^. Development partners and intergovernmental organisations could support this shift by benchmarking their investments in food systems against aims for an affordable, desirable, accessible supply of healthy food. Development partners could also review their portfolios across relevant sectors to ensure that these are promoting coherence with obesity prevention policy aims and not undermining them, and by introducing obesity prevention into all policy dialogue.

There is a specific opportunity to learn from the Pacific Islands Region to move forward on this challenge, where accountability systems have been crucial for building political will for NCD prevention and food systems reform^(18)^. Effective accountability would involve developing benchmarks that are appropriate to country context and therefore more compelling to national leaders^(10)^, with less emphasis on cross-context comparison. Accountability systems should be informed by participatory, scientific and traditional systems^(9,28)^ and take into account the capacity of countries to track and make use of such measures^(29)^. Developing the strategic capacities of health and political leaders to use accountability mechanisms that hold non-health sectors to account will help to ensure accountability measures are used in a more compelling way^(29)^.

### Conclusions

Monitoring and benchmarking are critical for overweight and obesity prevention, but the integration of food systems indicators will be essential to support action on the drivers of poor diets and obesity, in addition to ensuring that treatment needs are met. Effective accountability for obesity prevention should bring to account public and private institutions involved in the production, processing, transport, marketing, trade and consumption of food for their role in assuring an affordable, desirable, accessible healthy food supply. The mobilisation of food systems sectors towards obesity prevention will require a major paradigm shift such that those sectors are encouraged to carry an aligned sense of purpose and responsibility for their role, and are supported towards their commitments.
