# Why health systems cannot fix problems caused by food systems: a call to integrate accountability for obesity into food systems policy – CORRIGENDUM

**DOI:** 10.1017/S1368980024002441

**Published:** 2024-12-05

**Authors:** Erica Reeve, Penny Farrell, Anne Marie Thow, Senoveva Mauli, Dori Patay

The above article was originally published with a spelling error in Dori Patay’s name.

This has been corrected in the original article. The authors apologise for this error.
